# Prognostic implications of diastolic dysfunction in patients with acute pulmonary embolism

**DOI:** 10.1186/1756-0500-7-610

**Published:** 2014-09-06

**Authors:** Jae Hyung Cho, Roop Kaw, Jatin Chhabra, Snigdha Kola, Indrajeet Mahata, Shobha Shahani, Abraham G Kocheril

**Affiliations:** Department of Hospital Medicine, Cleveland Clinic, 9500 Euclid Avenue, M2-Annex, Cleveland, OH 44195 USA; Department of Hospital Medicine and Outcomes Research Anesthesiology, Cleveland Clinic, Cleveland, OH USA; Department of Hospital Medicine, Mercy Hospital, Springfield, MO USA; Department of Internal Medicine, University of Illinois at Urbana-Champaign, Urbana, IL USA; Department of Cardiology, University of Illinois at Urbana-Champaign, Urbana, IL USA

**Keywords:** Pulmonary embolism, Echocardiography, Heart failure, Diastolic

## Abstract

**Background:**

A history of congestive heart failure has been used to determine the prognosis in patients with acute pulmonary embolism. Diastolic dysfunction is responsible for the half of congestive heart failure but has not been understood well.

**Methods:**

A total of 205 patients were reported admitted with acute pulmonary embolism from January 2009 to July 2011. We excluded hemodynamically unstable patients who received thrombolytics or underwent thromboembolectomy. We included hemodynamically stable patients who underwent echocardiogram within 72 hours of diagnosis. We reviewed medical records of 107 patients to investigate whether diastolic dysfunction increases in-hospital mortality or adverse clinical outcomes.

**Results:**

Out of 107 patients, 10 patients died during hospitalization with in-hospital mortality rate of 9.3%. Among 84 patients without diastolic dysfunction as assessed by echocardiogram, six patients died with in-hospital mortality rate of 7.1%. Meanwhile, among 23 patients with diastolic dysfunction, four patients died with in-hospital mortality rate of 17.4%. The multivariable adjusted odds ratio was calculated as 2.71, with 95% confidence interval of 0.59 - 12.44.

**Conclusions:**

For hemodynamically stable patients with acute pulmonary embolism, diastolic dysfunction as assessed by echocardiogram could increase in-hospital mortality 2.71 fold, although this was not statistically significant. Further study with a large patient population is needed to determine the statistically significant implications of diastolic dysfunction in patients with acute pulmonary embolism.

## Background

Acute pulmonary embolism (PE) is a potentially life-threatening condition. Hemodynamically stable patients can be managed with anticoagulation alone, while hemodynamically unstable patients need thrombolytics or thromboembolectomy. Untreated patients can have a mortality rate of 30%, whereas treatment can lower the mortality rate down to 8% [1]. History of congestive heart failure (CHF) has been regarded as a negative prognostic factor according to the Pulmonary Embolism Severity Index (PESI) [2, 3].

CHF is a clinically diagnosed entity with characteristic symptoms and physical findings. Patients who have systolic or diastolic dysfunction on echocardiogram without CHF symptoms and signs, have preclinical systolic or diastolic dysfunction. Unlike systolic dysfunction, diastolic dysfunction has not been understood well although this comprises 40 to 71% of CHF [4, 5]. While we know that CHF can be a negative prognostic factor in patients with acute PE, the implications of diastolic dysfunction on patients with acute PE have not been studied before. We investigate whether diastolic dysfunction as assessed by echocardiogram can be used as a prognostic factor in hemodynamically stable patients with acute PE.

## Methods

### Study population

**Figure 1:**
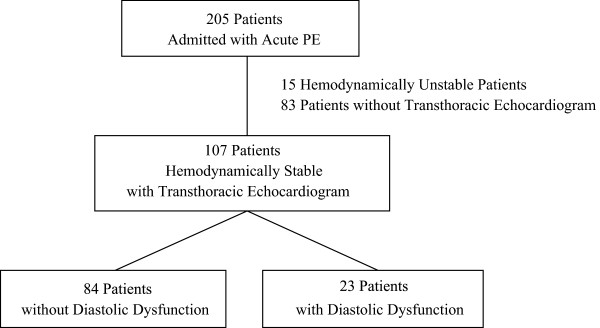
**Patient selection.**

We targeted hemodynamically stable patients with acute PE. A total of 205 patients were reported admitted with acute PE between January 2009 and July 2011. Our exclusion criteria were hemodynamically unstable patients who received thrombolytics or underwent thromboembolectomy. Our eligibility criteria were hemodynamically stable patients with systolic blood pressure of more than 90 mmHg who underwent echocardiogram within 72 hours of diagnosis. Out of 205 patients, 107 patients were selected meeting these criteria for our retrospective study (Figure 1). We reviewed medical records to investigate whether diastolic dysfunction as assessed by echocardiogram increases in-hospital mortality or adverse clinical outcomes such as shock, need for vasopressor, intubation or cardiopulmonary resuscitation in patients with acute PE. All of these 107 patients were managed with intravenous heparin infusion or subcutaneous enoxaparin.

### Diagnosis of pulmonary embolism

Patients were diagnosed with acute PE with either computed tomography or ventilation/perfusion lung scan. Out of 107 patients, 100 patients (93.5%) were diagnosed with computed tomography while 7 patients (6.5%) were diagnosed with ventilation/perfusion lung scan.

### Echocardiogram

We only included patients with acute PE who had echocardiogram within 72 hours of diagnosis. Diagnosis of diastolic dysfunction was made with the following characteristics: mild (impaired relaxation without increased filling pressure), moderate (impaired relaxation with moderate elevation of filling pressure or pseudo-normal filling pressure) and severe (advanced reduction in compliance or reversible or fixed restrictive filling). E/A, E/e’ and deceleration time were used to classify diastolic dysfunction as mild, moderate and severe (E: peak early filling velocity, A: velocity at atrial contraction, e’: velocity of mitral annulus early diastolic motion). Normal diastolic function was defined as 0.75 < E/A < 1.5, deceleration time > 140 ms and E/e’ < 10; mild diastolic dysfunction was characterized with E/A < 0.75 and E/e’ < 10; moderate diastolic dysfunction was described as 0.75 < E/A < 1.5, deceleration time > 140 ms and E/e’ > 10; severe diastolic dysfunction was defined as E/A > 1.5, deceleration time < 140 ms and E/e’ > 10 [6]. For patients with atrial fibrillation, E/e’ and deceleration time were used to classify diastolic dysfunction since E/A could not be measured [7–9].

### Study outcome

The primary study outcome was in-hospital death and secondary study outcomes were adverse clinical events such as shock, need for vasopressor, intubation and cardiopulmonary resuscitation. Since we already excluded hemodynamically unstable patients, the adverse clinical outcomes of shock or need for vasopressor indicate in-hospital adverse outcomes.

### Statistical analysis

We used SPSS 19 for the statistical analysis. Differences in baseline patient characteristics were compared using the independent samples *T*-test for continuous variables and chi-square test for categorical variables. Unadjusted and multivariable adjusted odds ratio and P value were calculated using binary logistic regression.

### Institutional review board

This study was approved by the Institutional Review Board at the University of Illinois at Urbana-Champaign, # 11095. There was no ethical issue in our study since this is a retrospective medical record review.

### Funding

There was no funding used for the performance of this study.

## Results

### Baseline patient characteristics

A total of 205 patients were admitted with the diagnosis of acute PE between January 2009 and July 2011. After excluding hemodynamically unstable patients who received thrombolytics or underwent thromboembolectomy and patients who did not undergo echocardiogram within 72 hours of diagnosis, 107 patients were selected for our retrospective study (Figure 1). Most of the baseline characteristics were not statistically different between the two groups except for age and hypertension (Table 1). Patients with diastolic dysfunction were significantly older than patients without diastolic dysfunction (67.0 ± 17.0 vs. 58.1 ± 18.6, P value 0.040). Patients with diastolic dysfunction had baseline hypertension significantly more than patients without diastolic dysfunction (86.9% vs. 58.3%, P value 0.011). Of note, there was no difference in EF between the two groups (59.1 vs. 57.4%, P value 0.491).

**Table 1:** **Baseline patient characteristics**

Characteristics (107 patients)	Patients without diastolic dysfunction (84 patients, 78.5%)	Patients with diastolic dysfunction (23 patients, 21.5%)	P value
**Age**	58.1 ± 18.6	67.0 ± 17.0	0.040*
**Male sex**	43 (51.2%)	13 (56.5%)	0.650
**Hypertension**	49 (58.3%)	20 (86.9%)	0.011*
**Diabetes mellitus**	24 (28.6%)	7 (30.4%)	0.861
**Hyperlipidemia**	39 (46.4%)	10 (43.5%)	0.801
**CAD**	23 (27.4%)	5 (21.7)	0.623
**COPD**	13 (15.5%)	2 (8.7%)	0.407
**CKD**	6 (9.5%)	2 (8.7%)	0.802
**CHF**	8 (9.5%)	4 (17.4%)	0.289
**Ejection fraction**	59.1 ± 11.1	57.4 ± 8.0	0.491
**Atrial fibrillation**	15 (17.9%)	3 (13.0%)	0.585
**Peripheral vascular disease**	5 (6.0%)	3 (13.0%)	0.252

*indicates statistically significant result of P value < 0.05.

### History of congestive heart failure

The history of CHF was obtained from medical records when the patient has clinically diagnosed CHF before developing acute PE. Eight patients had a diagnosis of CHF before developing PE among 84 patients without diastolic dysfunction (9.5%), and four patients had a diagnosis among 23 patients with diastolic dysfunction (17.4%). Most of the patients had systolic CHF except for one patient who had documented diastolic CHF.

### Diastolic dysfunction on echocardiogram

The prevalence of diastolic dysfunction on echocardiogram in 107 patients with acute PE was 21.5% (23 patients out of 107 patients). We looked at previous echocardiogram to find out the acuity of diastolic dysfunction but the data were limited. The degree of diastolic dysfunction was mild for 12 patients, moderate for 10 patients and severe for 1 patient (Table 2).

**Table 2:** **In-hospital mortality (degree of diastolic dysfunction)**

	Patients without diastolic dysfunction (84, 78.5%)	Patients with diastolic dysfunction (23, 21.5%)		
		Mild (12, 11.2%)	Moderate (10, 9.3%)	Severe (1, 1.0%)
**In-hospital death**	6 (7.1%)	2 (16.7%)	2 (20%)	0 (0%)

### In-hospital mortality

The primary study outcome was in-hospital death. Out of 107 patients, 10 patients died during hospitalization with in-hospital mortality rate of 9.3%. The cause of death for these 10 patients was pulmonary embolism. Among 84 patients without diastolic dysfunction as assessed by echocardiogram, six patients died with in-hospital mortality rate of 7.1%. Meanwhile, among 23 patients with diastolic dysfunction as assessed by echocardiogram, four patients died with in-hospital mortality rate of 17.4%. Based on degree of diastolic dysfunction, in-hospital mortality was calculated as 16.7% for mild diastolic dysfunction, 20% for moderate diastolic dysfunction and 0% for severe diastolic dysfunction (Table 2). The unadjusted odds ratio for diastolic dysfunction was calculated as 2.74 with 95% confidence interval of 0.70 - 10.67 (Table 3). The adjusted odds ratio for age and hypertension was calculated as 2.71 with 95% confidence interval of 0.59 - 12.44 (Table 3).

**Table 3:** **In-hospital mortality (without vs. with diastolic dysfunction)**

	Patients without diastolic dysfunction (84, 78.5%)	Patients with diastolic dysfunction (23, 21.5%)	Unadjusted odds ratio (95% confidence interval)	Adjusted* odds ratio (95% confidence interval)
**In-hospital death**	6 (7.1%)	4 (18.2%)	2.74 (0.70 – 10.67)	2.71 (0.59 – 12.44)

*Adjusted for age and hypertension.

### Adverse clinical outcomes

We also looked at adverse clinical events as the secondary outcomes. Adverse clinical outcomes included shock, need for vasopressor, intubation and cardiopulmonary resuscitation during hospitalization. The complications of shock and need for vasopressor were more associated with patients without diastolic dysfunction although these were not statistically significant (odds ratio for shock 0.21 with 95% confidence interval of 0.03 - 1.67 and 0% vs. 6% for need for vasopressor) (Table 4). There was no difference for need for intubation between two groups (odds ratio 1.24 with 95% confidence interval 0.23 - 6.59). The need for cardiopulmonary resuscitation was observed in 4.8% (patients without diastolic dysfunction) vs. 0% (patients with diastolic dysfunction).

**Table 4:** **Adverse clinical outcomes**

	Patients without diastolic dysfunction (84, 78.5%)	Patients with diastolic dysfunction (23, 21.5%)	Odds ratio (95% confidence interval)
**Shock**	15 (17.9%)	1 (4.3%)	0.21 (0.03 – 1.67)
**Vasopressor**	5 (6%)	0 (0%)	N/A
**Intubation**	6 (7.1%)	2 (8.7%)	1.24 (0.23 – 6.59)
**CPR**	4 (4.8%)	0 (0%)	N/A

N/A: Non-Available.

## Discussion

A history of CHF is regarded as one of negative prognostic factors in patients with acute PE. Although diastolic dysfunction comprises significant portion of CHF, the implications of diastolic dysfunction for predicting mortality in patients with acute PE has not been investigated before. We investigated whether diastolic dysfunction increases in-hospital mortality or adverse clinical outcomes in patients with acute PE. Our study showed that, for hemodynamically stable patients with acute PE, diastolic dysfunction as assessed by echocardiogram could increase in-hospital mortality 2.71 fold, although this was not statistically significant.

Since we do not have baseline echocardiography for most of the patients, we cannot determine whether this is a chronic manifestation due to preclinical CHF or an acute finding due to PE. There were several studies in the past which investigated abnormal left ventricular diastolic function in patients with chronic thromboembolic hypertension, which revealed that the low left ventricular preload and relative under-filling can cause diastolic dysfunction in patients with chronic PE [10, 11]. So it is possible that acute PE can cause diastolic dysfunction by the low preload and under-filling of the left ventricle. Based on a previous study of Olmsted County, the prevalence of diastolic dysfunction was reported as 20.8% for mild dysfunction, 6.6% for moderate dysfunction and 0.7% for severe dysfunction [6]. Our study showed the prevalence of diastolic dysfunction in patients with acute PE of 21.5%, which supports the likelihood that diastolic dysfunction was more likely a chronic condition. Nonetheless, we can safely say that patients with diastolic dysfunction on echocardiogram have an increased mortality compared to patients without diastolic dysfunction.

As shown in Table 3, adverse clinical outcomes varied. The incidence of shock and need for vasopressor were low in patient with diastolic dysfunction compared to patients without diastolic dysfunction. Patients with diastolic dysfunction were found to be more hypertensive than patients without diastolic dysfunction as shown in Table 1. This is probably the reason for the low rate of shock and need for vasopressor in patients with diastolic dysfunction. There was no difference in terms of need for intubation or cardiopulmonary resuscitation between the two groups. Unfortunately all the results were not statistically significant and probably this is because of low number of patients included in this analysis.

There are a few limitations in our retrospective study. First of all, the increase of the mortality by 2.71 times is not statistically significant. Although 7.1% vs. 17.4% could be a striking difference, because of the small number of patients included, we could not show a statistically significant result. Further study with a large patient population is needed to determine the statistically significant difference. Second, there were two differences in baseline patient characteristics; age and hypertension. Both of them are regarded as risk factors for diastolic dysfunction. We already know that age is a negative prognostic factor in patients with acute PE and diastolic dysfunction could be dependent on old age. Third, we only included hemodynamically stable patients with systolic blood pressure more than 90 mmHg. We did not investigate hemodynamically unstable patients who received thrombolytics or underwent thromboembolectomy. It could be possible that patients with diastolic dysfunction were more hemodynamically unstable and thus excluded from our study when they got benefit from thrombolytics or thromboembolectomy. This remains a future research area.

Our research was a retrospective study of hemodynamically stable patients with acute PE to see whether diastolic dysfunction predicts increased in-hospital mortality or adverse clinical outcomes. Even though this is a retrospective study involving a small number of patients, we reached an important finding. Further study is needed with a large number of patients to reach a statistically significant result and to investigate the benefit of thrombolytics in patients with diastolic dysfunction.

## Conclusions

For hemodynamically stable patients with acute pulmonary embolism, diastolic dysfunction as assessed by echocardiogram could increase in-hospital mortality 2.71 fold, although this was not statistically significant. Further study with a large patient population is needed to determine the statistically significant implications of diastolic dysfunction in patients with acute pulmonary embolism.

## Abbreviations

PE: Pulmonary Embolism
CHF: Congestive Heart Failure
EF: Ejection Fraction
PESI: Pulmonary Embolism Severity Index
E/A: E: peak early filling velocity, A: velocity at atrial contraction
E/e’: E: peak early filling velocity, e’: velocity of mitral annulus early diastolic motion.
